# Homocysteine accelerates hepatocyte autophagy by upregulating TFEB via DNMT3b-mediated DNA hypomethylation: TFEB accelerates autophagy of hepatocytes with Hcy treatment

**DOI:** 10.3724/abbs.2023060

**Published:** 2023-04-06

**Authors:** Anning Yang, Wen Zeng, Hongwen Zhang, Yinju Hao, Qingqing Wang, Yue Sun, Shangkun Quan, Ning Ding, Xiaoling Yang, Jianmin Sun, Huiping Zhang, Bin Liu, Yun Jiao, Kai Wu, Yideng Jiang

**Affiliations:** 1 College of Biology Hunan University Changsha 41000 China; 2 General Hospital of Ningxia Medical University Yinchuan 750004 China; 3Department of Pathophysiology National Health Commission Key Laboratory of Metabolic Cardiovascular Diseases Research School of Basic Medical Sciences Ningxia Medical University Yinchuan 750004 China; 4 Ningxia Key Laboratory of Vascular Injury and Repair Research Ningxia Medical University Yinchuan 750004 China; 5 Translational Cancer Research Department of Laboratory Medicine Lund University Lund Sweden; 6 Hunan Provincial Maternal and Child Health Care Hospital Changsha 410000 China; 7 School of Public Health and Management Ningxia Medical University Yinchuan 750004 China; 8 Department of Scientific Research and Teaching Central Hospital of Shaoyang Shaoyang 422000 China

**Keywords:** homocysteine, TFEB, autophagy, DNA methylation, DNA methyltransferase 3b

## Abstract

Autophagy plays a critical role in the physiology and pathophysiology of hepatocytes. High level of homocysteine (Hcy) promotes autophagy in hepatocytes, but the underlying mechanism is still unknown. Here, we investigate the relationship between Hcy-induced autophagy level and the expression of nuclear transcription factor EB (TFEB). The results show that Hcy-induced autophagy level is mediated by upregulation of TFEB. Silencing of *TFEB* decreases the level of autophagy-related protein LC3BII/I and increases p62 expression level in hepatocytes after exposure to Hcy. Moreover, the effect of Hcy on the expression of TFEB is regulated by hypomethylation of the *TFEB* promoter catalyzed by DNA methyltransferase 3b (DNMT3b). In summary, this study shows that Hcy can activate autophagy by inhibiting DNMT3b-mediated DNA methylation and upregulating TFEB expression. These findings provide another new mechanism for Hcy-induced autophagy in hepatocytes.

## Introduction

Homocysteine (Hcy) is an intermediate of methionine metabolism [1]. Dysregulation of homocysteine metabolism leads to hyperhomocysteinaemia (HHcy), an independent risk factor for cardiovascular and cerebrovascular diseases [2]. The liver is the main organ for Hcy metabolism; however, liver injury, such as chronic liver disease, liver cirrhosis, or even primary liver cancer, results in an increased level of Hcy in circulation [ 3‒ 5] .

The transcription factor EB (TFEB) is a downstream regulatory factor of rapamycin target protein complex 1 (mTORC1) and is negatively modulated by mTORC1 [6]. It plays a vital role in lysosomal biosynthesis, autophagy, and angiogenesis and promotes lipid degradation *in vivo* [7]. Under physiological conditions, TFEB is inactive in the cytosol, whereas fasting and stress stimuli induce its dephosphorylation-mediated activation and subsequent nuclear translocation. Previous studies have shown that TFEB is the primary regulator of autophagy [8]. TFEB actively regulates autophagy, activates lysosomal genes, promotes the formation and fusion of autophagosomes and lysosomes, increases the process of autophagic flux, and enhances the ability of cells to degrade lysosomal substrates [9]. Moreover, TFEB also enhances lipid decomposition and liver lysosomal enzyme activity in a fulminant hepatitis mouse model [10]. However, whether TFEB mediates the effect of Hcy on hepatocyte autophagy remains unclear.

DNA methylation, as a type of epigenetic regulation, inhibits DNA methyltransferase-mediated gene expression [ 11, 12] . Hcy is a methyl donor and is involved in gene expression changes by DNA methylation [13]. Meanwhile, HHcy in mammals regulates the expressions and activities of DNA methyltransferases (DNMTs), including DNMT1, DNMT3a, and DNMT3b. For instance, cystathionine-beta-synthase deficiency mouse-induced HHcy leads to a significant increase in DNMT activity [14]. Similarly, Hcy promotes DNMT1 protein expression in human umbilical vein endothelial cells [15]. DNMT1 and DNMT3a expressions were elevated, while DNMT3b expression was decreased in Hcy-induced mouse brain endothelial cells [16]. However, whether DNA methylation affects the expression of Hcy-induced TFEB and the exact mechanisms have not been fully elucidated.

In the present study, we investigated the precise regulatory role and mechanism of TFEB in Hcy-induced autophagy in hepatocytes. Our results revealed that TFEB is an important regulatory mediator of autophagy, and its expression is modulated by DNA methylation. Our findings provide novel insights into the molecular mechanism uderlying Hcy-induced autophagy in hepatocytes.

## Materials and Methods

### Materials

L-Hcy (6943) and the DNA methylation inhibitor 5-azacytidine (AZC; A23885) were obtained from Sigma-Aldrich (St Louis, USA). DC-05 (HY-12746), Theaflavin-3,3′-digallate (TF-3; HY-N1992), and Nanaomycin A (NA; HY-103397) were purchased from MedChemExpress (Monmouth, USA).

### Cell culture

The human hepatocyte line (HL-7702) was purchased from the Cell Bank of the Chinese Academy of Sciences (Shanghai, China) and cultured in RPMI-1640 medium (Thermo Fisher Scientific, Waltham, USA) containing 8% fetal bovine serum (FBS; HyClone, Carlsbad, USA), streptomycin (100 μg/mL) and penicillin (100 U/mL). When cells reached 80% confluence, subsequent experiments were performed after 24 h of intervention with L-Hcy (100 μM).

### Quantitative real-time PCR (qRT-PCR)

qRT-PCR analysis was performed as described previously [17]. Total RNA was extracted using a commercial kit (Takara, Dalian, China) according to the manufacturer’s protocol. Single-strand cDNA was generated with a SuperScript™ IV One-Step RT-PCR System (Thermo Fisher Scientific). qRT-PCR was performed using PowerTrack™SYBR Green Master Mix (Thermo Fisher Scientific) on an ABI7500 real-time PCR system (Applied Biosystems, Foster City, USA). Glyceraldehyde phosphate dehydrogenase ( *GAPDH*) served as an internal reference gene. Relative quantification of mRNA expression was calculated using the 2 ^‒△△Ct^ method. The primer sequences are shown in Table 1.

**Table:** **Table 1** Sequences of primers used for qRT-PCR analysis

Gene	GenBank	Primer sequence (5′→3′)	Product size (bp)	Tm (°C)
*TFEB*	NM_007162.3	F: TGATGGTGAACTCGCAAGAAG R: TATATGTTCACTGCGTCCTCCG	120	58.58
*DNMT3b*	NM_006892.4	F: CAGCTGCTCCCGGCTC R: TACTTGGGCCACTTAACCCC	140	59.30
*GAPDH*	NM_002046.7	F: CTCTGCTCCTCCTGTTCGAC R: GCGCCCAATACGACCAAATC	121	59.83

F: forward primer; R: reverse primer.

### Western blot analysis

Hepatocytes were lysed with lysis buffer supplemented with phenyl methane sulfonyl fluoride (PMSF) as previously described [17]. After centrifugation, protein concentrations in the extracts were analyzed using the BCA assay kit (Beyotime Institute of Biotechnology, Shanghai, China). Proteins (30 μg from each extract) were separated by SDS-PAGE and then transferred onto polyvinylidene fluoride (PVDF) membranes (Millipore, Billerica, USA). Membranes were blocked with 5% defatted milk and then incubated with primary antibodies against DNMT1 (PA5-30581; Thermo Fisher Scientific), TFEB (ab267351; Abcam, Cambridge, USA), DNMT3a (ab2850; Abcam), DNMT3b (ab2851; Abcam), p62 (ab109012; Abcam), microtubule-associated protein 1 light chain 3 (LC3; ab192890; Abcam), and β-actin (ab8226; Abcam) respectively, followed by incubation with horseradish peroxidase (HRP)-labelled secondary antibody. Finally, enhanced chemiluminescence solution (Millipore) was used for detection of the indicated proteins, and immunoblot images were analysed with ChemiDoc (Bio-Rad, Hercules, USA).

### Nested methylation-specific polymerase chain reaction (nMS-PCR)

Genomic DNA was isolated from the hepatocytes using the WizardÒGenomic DNA Purification kit (Promega, Madison, USA). This integrated the DNA denaturation and bisulfite conversion processes into one step by the EZ DNA Methylation-Gold ^TM^ kit (ZYMO Research, Orange County, USA). The eluted DNA sample was stored at 20°C. In the first step of nMS-PCR, an outer primer pair set that did not include any CpG was used. The second-step PCR was performed with conventional PCR primers. The sequences of the primers used for the nMS-PCR assays are shown in Table 2. PCR products were purified with an agarose gel. To reduce misprinting and increase efficiency, touchdown (TD) PCR was used for amplification. Samples were subjected to 25 cycles in a TD program (95°C for 30 s, 56.5°C for 30 s, and 72°C for 1 min), followed by a 0.5°C decrease in the annealing temperature every cycle. After completion of the TD program, 20 cycles were subsequently run (95°C for 35 s, 50°C for 35 s and 72°C for 35 s), ending with a 3 min extension at 72°C. The PCR products were separated by electrophoresis using a 2% agarose gel containing ethidium bromide. DNA bands were visualized by ultraviolet light. The presence of methylation was calculated using the following formula: methylation (%)=methylation/(methylation+unmethylation)×100%.

**Table:** **Table 2** nMS-PCR primers for TFEB

Primer set	Primer sequence (5′→3′)	Product size (bp)	Tm (°C)
TFEB-O	F: GGTGTTATGGATAAAAAGAGGAAGTT R: CCACTACAATCACAACCTCAAAATA	256	59.3
TFEB-M	F: TGTTATGTGGTAGATATTGGTTCGT R: TAAACTCTATAACGCTCAACACGTC	119	58.5
TFEB-U	F: TGTTATGTGGTAGATATTGGTTTGT R: CTCTAAACTCTATAACACTCAACACATC	121	56.2

O, out primer; M, methylation primer; U: unmethylation primer; F: Forward primer; R: Reverse primer.

### Construction of recombinant DNMT3b adenovirus

Recombinant adenoviruses expressing the human *DNMT3b* gene were constructed with the replication-defective adenoviral shuttle vector pHBAd-CMV-IRES-GFP and the adenoviral backbone plasmid pBHGlox(Delta)E1,3Cre. The DNMT3b fragments were inserted into the pHBAd-CMV-IRES-GFP vector and cotransfected with pBHGlox(Delta)E1,3Cre into the virus packaging cell line 293. Recombinant adenoviruses were expanded, purified, collected, and used to infect the liver cell line HL-7702. The recombinant adenovirus encoding green fluorescent protein (Ad-GFP) was used as a control. Hepatocytes at approximately 80% confluence were infected with purified adenovirus for further experiments. Western blot analysis was used to detect ectopic gene expression using antibodies against DNMT3b.

### Construction of TFEB and DNMT3b shRNA adenovirus

For the knockdown of *TFEB* and *DNMT3b* by short hairpin (shRNA), shRNA adenoviral particles were purchased from Genepharma (Shanghai, China) and packaged into HEK293 cells according to the manufacturer’s guidelines. The sequences of TFEB and DNMT3b shRNA are listed in Table 3.

**Table:** **Table 3** Sequences of shRNAs against TFEB and DNMT3b

shRNA	Sequence (5′→3′)
TFEB-1	CCGGCCCACTTTGGTGCTAATAGCTCTCGAGAGCTATTAGCACCAAAGTGGGTTTTT
TFEB-2	CCGGCGATGTCCTTGGCTACATCAACTCGAGTTGATGTAGCCAAGGACATCGTTTTT
TFEB-3	CCGGGAGACGAAGGTTCAACATCAACTCGAGTTGATGTTGAACCTTCGTCTCTTTTT
DNMT3b-1	CCGGGCCTCAAGACAAATTGCTATACTCGAGTATAGCAATTTGTCTTGAGGCTTTTTG
DNMT3b-2	CCGGGCCCGTGATAGCATCAAAGAACTCGAGTTCTTTGATGCTATCACGGGCTTTTTG
DNMT3b-3	CCGGCCATGCAACGATCTCTCAAATCTCGAGATTTGAGAGATCGTTGCATGGTTTTTG

### MassARRAY DNA methylation analysis

Quantitative analysis of differentially methylated regions was performed by BioMiao Biological Technology (Beijing, China) with the Agena MassARRAY platform (Agena, San Diego, USA). Briefly, genomic DNA was isolated from HL-7702 cell lines, and 2 μg DNA was treated with sodium bisulfite using an EZ DNA Methylation-Gold kit (ZYMO Research) according to the manufacturer’s instructions. The specific primers based on the reverse complementary strands of the *TFEB* promoter were designed using EpiDesigner software (Agena), and the quantitative results for each CpG or multiple CpGs were analyzed with EpiTYPER ^TM^ (Agena). The primer sequences were 5′-aggaagagagAGGTATTTAAGGGTATTTTTGGTGG-3′ and 3′-cagtaatacgactcactatagggagaaggctCCTATAATCCCAACATTTTAAAAAACC-5′. Bisulfite-modified DNA PCR amplifications were performed with a precycling hold at 94°C for 4 min and subjected to 45 cycles of 94°C for 20 s, 56°C for 20 s,72°C for 1 min, and a final extension at 72°C for 3 min. Further experimental analysis of the contents of DNA methylation was determined, as described previously [18].

### mRFP-GFP-LC3 fluorescence microscopy

mRFP-GFP-LC3 adenoviral vectors were obtained from HanBio Technology (Shanghai, China). Hepatocyte autophagy was analysed using tandem mRFP-GFP-LC3 fluorescence microscopy. Hepatocytes were cultured in 35-mm confocal dishes for 24 h and then infected with mRFP-GFP-LC3 adenovirus for 2 h. Then, 100 μM Hcy was added to the medium and incubated for 24 h at 37°C. Yellow and red puncta were observed using a BX51 confocal fluorescence microscope (Olympus, Tokyo, Japan).

### Immunofluorescence microscopy

Hepatocytes were fixed with 4% paraformaldehyde for 20 min at room temperature (RT), permeabilized with 0.1% Triton on ice, and then blocked in PBS containing 5% bovine serum albumin (BSA) for 1 h, followed by incubation with a specific antibody against TFEB (ab267351; Abcam) overnight at 4°C. After that, Alexa Fluo-conjugated secondary antibody (Life Technologies, Waltham, USA) and DAPI were applied for 2 h at RT. Cells were then imaged with a BX51 laser confocal microscope (Olympus).

### Chromatin immunoprecipitation (ChIP) assays

Formaldehyde at 1% was applied to the hepatocytes for cross-linking. After 15 min, glycine was added to a maximum concentration of 125 mM. Cells were washed with cold PBS, collected, and then sonicated on a 20% power ultrasonic lyser to cut the DNA into fragments with an average size of 200 to 1000 bp. Then, 50 μL from each sonicated sample was pipetted, and the fragment size and DNA concentration were measured. Cell lysates were incubated overnight with 30 μL ChIP-grade Protein G agarose beads (Millipore) and 10 μg antibodies against DNMT1, DNMT3a, or DNMT3b (Abcam). Agarose beads were collected and sequentially exposed to proteinase K at 60°C for 3 h and RNase at 37°C for 2 h. DNA purification was performed using a SpinPrep™ PCR Clean-up kit (Millipore). DNA fragments were assayed by qRT-PCR using the following primer sequences: forward primer: 5′-CTGGTATTAGCCAGAACATGTCAG-3′, and reverse primer: 5′-CCTCTTGCACAGTATGTAGCACC-3′. Samples were standardized to input DNA.

### Statistical analysis

Data are presented as the mean±standard deviations (SD), and statistical significance was analyzed with one-way analysis of variance (ANOVA), followed by the Newman-Keuls test. Each test was carried out three times. Significance was defined as a *P* value<0.05, unless otherwise stated.

## Results

### Hcy promotes autophagy in hepatocytes

**Figure 1:**
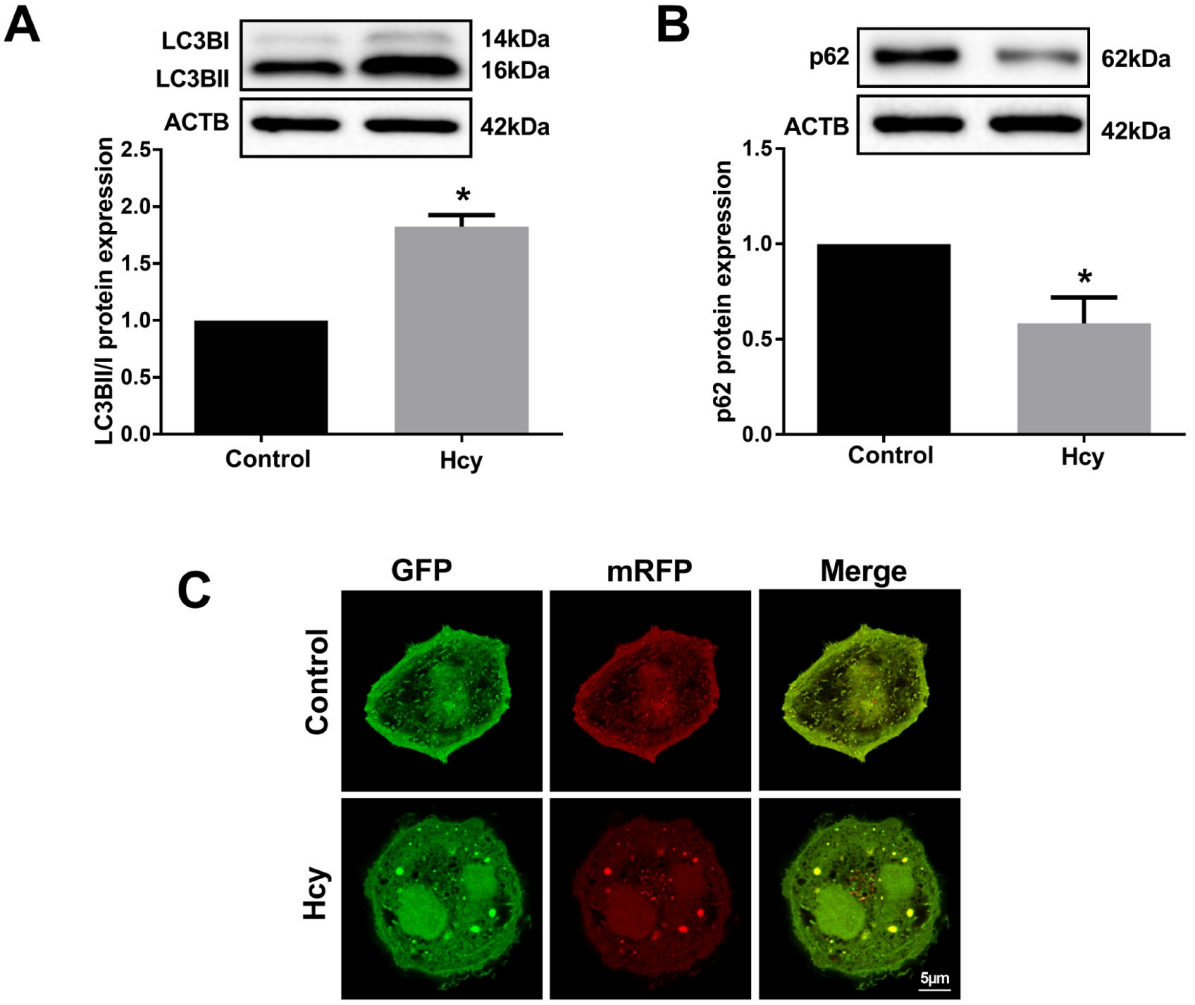
Figure 1 Autophagy assay in Hcy-induced hepatocytes (A,B) The protein levels of LC3BII/I and p62 in Hcy-induced hepatocytes were measured by western blot analysis (Hcy: 100 μM). (C) Tandem mRFP-GFP-LC3 fluorescence microscopy was performed to analyze hepatocyte autophagy. Liver cells were incubated with Hcy (100 μM) for 24 h (scale bar: 5 μm). Yellow puncta, the merge of mRFP and GFP fluorescence, indicate autophagosomes, whereas free red puncta (mRFP only) represent autolysosomes. Data are presented as the mean±SD from three independent experiments, * P<0.05.

We used 100 μM Hcy to stimulate hepatocytes to verify whether Hcy induces hepatocyte autophagy. We examined the conversion of LC3BI to LC3BII (a marker for autophagy activation) and p62 expression (a marker of autophagy inhibition), as well as the initiation of macroautophagy flux. The results showed that Hcy increased the ratio of LC3BII/I and inhibited p62 expression in hepatocytes ( Figure 1A,B). Interestingly, hepatocytes were infected with a pH-sensitive tandem mRFP-GFP-LC3 adenovirus to monitor autophagy-induced puncta formation. Yellow puncta reflect the combination of mRFP and GFP fluorescence labels, which represent autophagosomes, whereas free red puncta reflect only mRFP labels, which represent autolysosomes due to the quenching of GFP fluorescence under acidic pH conditions. As shown in Figure 1C, free red and yellow puncta were significantly increased in hepatocytes, indicating an increase in both autophagosomes and autolysosomes. Collectively, these data indicate that Hcy induces autophagy in hepatocytes.

### TFEB plays an essential role in promoting hepatocyte autophagy induced by Hcy

**Figure 2:**
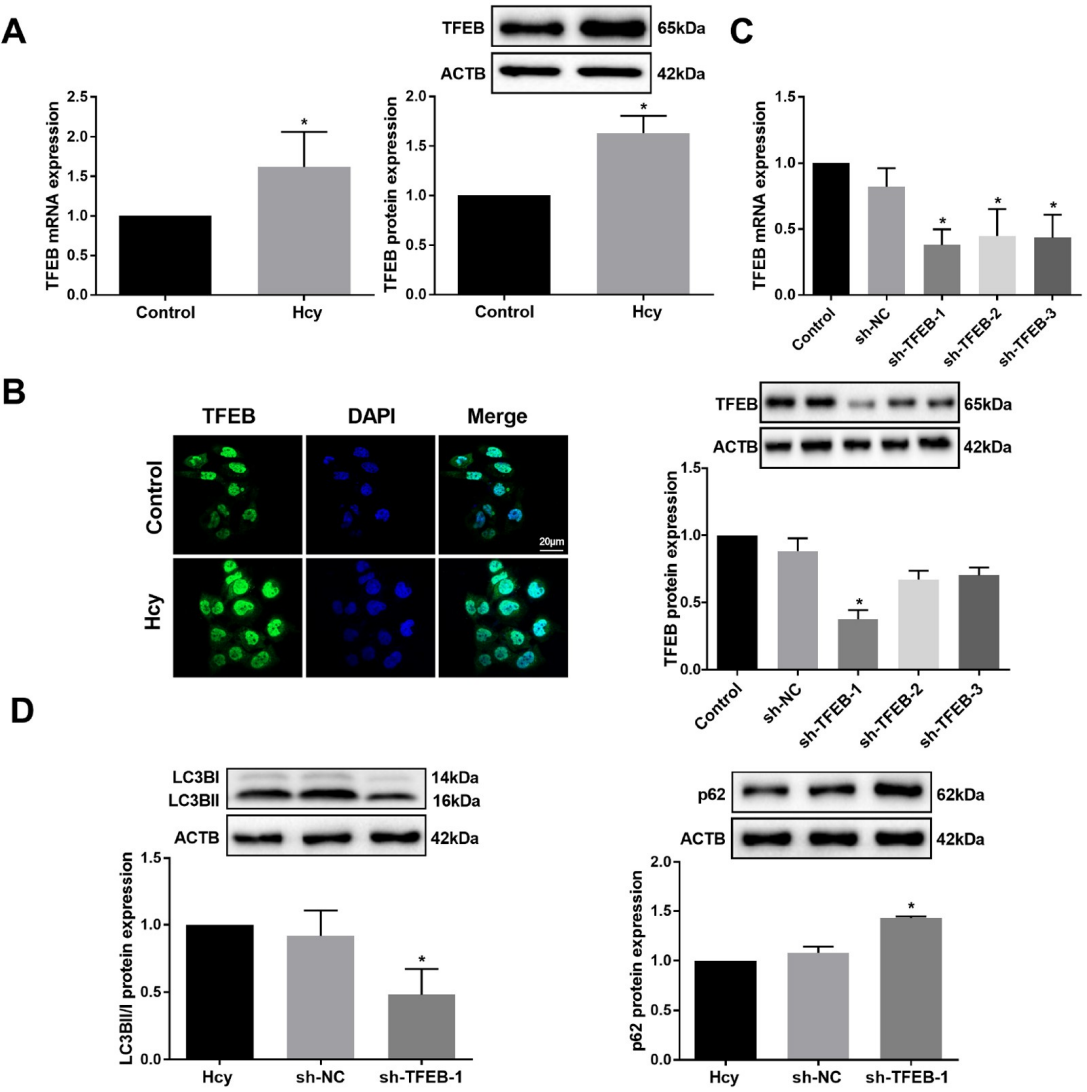
Figure 2 Hcy promotes autophagy by upregulating TFEB levels in hepatocytes (A) TFEB mRNA and protein expressions in hepatocytes were measured by qRT-PCR and western blot analysis, respectively. (B) Immunofluorescence staining of TFEB (green) in hepatocytes after treatment with Hcy. The nuclei were stained with DAPI (blue) (scale bar: 20 μm). (C) mRNA and protein levels of TFEB were measured by qRT-PCR and western blot analysis, respectively in hepatocytes after infection with sh-TFEB adenovirus (sh-TFEB-1, sh-TFEB-2, sh-TFEB-3) or sh-NC (control). (D) The protein expressions of LC3BII/I and p62 were detected by western blot analysis in Hcy-induced hepatocytes infected with sh-TFEB-1 adenovirus. Data are presented as the mean±SD from three independent experiments, * P<0.05.

To determine whether TFEB is involved in Hcy-induced liver autophagy, TFEB mRNA and protein expressions were analyzed by qRT-PCR and western blot analysis respectively in hepatocytes exposed to Hcy. We found that TFEB expression was increased in hepatic cells exposed to Hcy ( *P*<0.05; Figure 2A). Additionally, laser confocal microscopy showed that TFEB was primarily distributed in the nucleus. The fluorescence signal intensity in hepatocytes exposed to Hcy was stronger than that in the control, indicating that Hcy promoted the expression of TFEB ( Figure 2B). Then, TFEB shRNA adenovirus (sh-TFEB) was infected into hepatocytes to verify its interference efficiency ( Figure 2C). We found that knockdown of *TFEB* inhibited the proportions of LCB3II/I and enhanced the expression of p62 in hepatocytes ( Figure 2D). These results indicate that TFEB plays an important role in Hcy-induced autophagy in hepatocytes.

### TFEB hypomethylation plays an essential role in upregulating TFEB expression in hepatocytes

**Figure 3:**
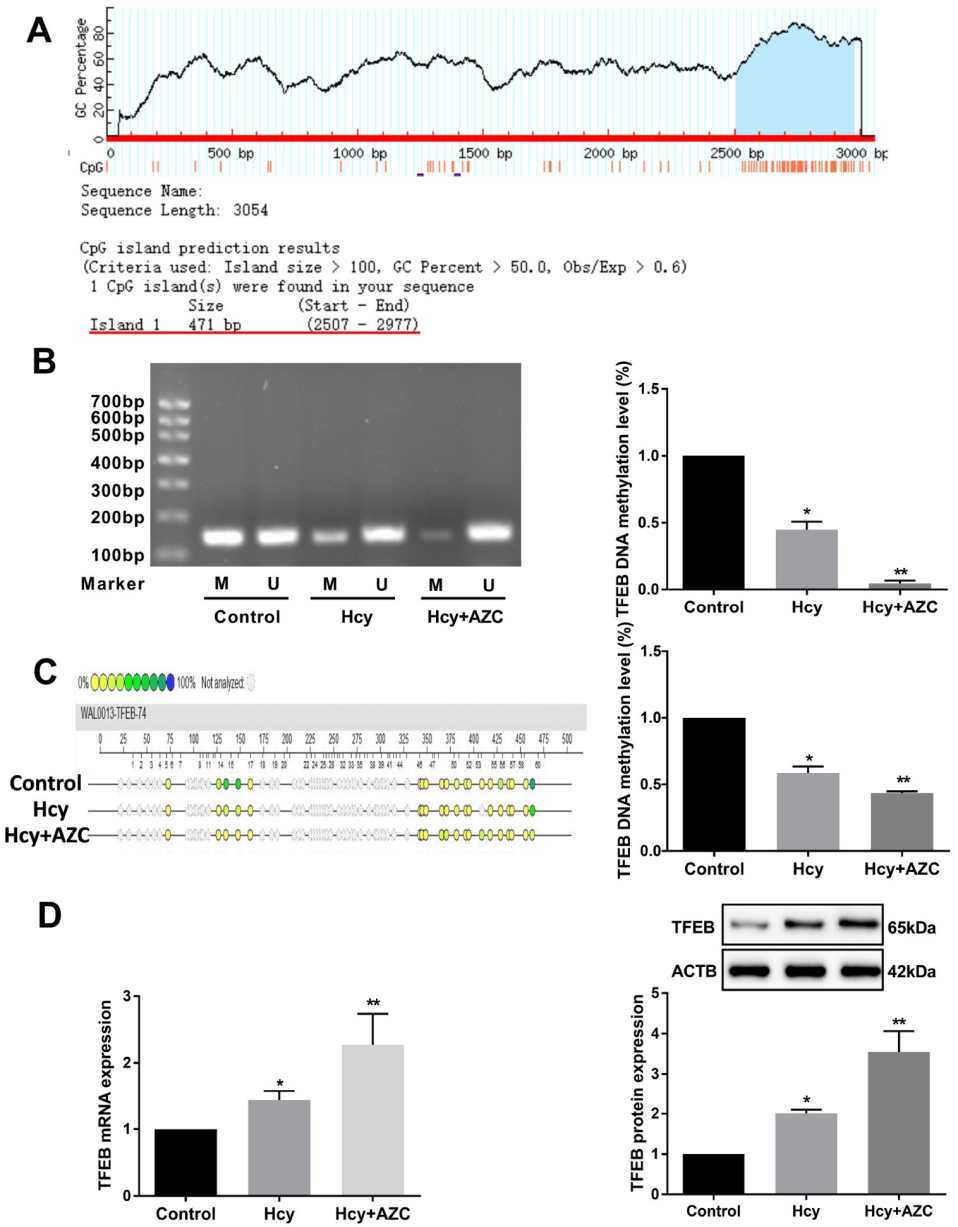
Figure 3 Hcy downregulates the level of TFEB DNA methylation (A) Screenshots of the putative CpG islands in the 5′-flank regions of the TFEB gene. (B) The level of DNA methylation in the TFEB promoter was detected by nMS-PCR in hepatocytes after exposure to Hcy or Hcy plus 5-azacytidine (AZC). M indicates the methylated band; U indicates the unmethylated band. (C) MassARRAY was used to examine the methylation status of the TFEB promoter in hepatocytes. The circle color represents the percentage of methylation in each CpG site. Blue indicates methylation (100%), and yellow indicates a lack of methylation (0%). Gray circles represent the unanalyzed CpG sites. (D) The mRNA and protein expressions of TFEB were measured by qRT-PCR and western blot analysis, respectively in hepatocytes exposed to Hcy and AZC. Data are presented as the mean±SD from three independent experiments, * P<0.05, ** P<0.01.

To investigate whether Hcy modulates TFEB expression via CpG methylation, we used MethPrimer software to predict CpG island changes (+483 bp/ +953 bp) in the *TFEB* promoter region ( Figure 3A). The methylation status of CpG islands in the *TFEB* promoter was measured by nMS-PCR and MassARRAY ( Figure 3B,C). As expected, Hcy reduced DNA methylation of the *TFEB* promoter in hepatocytes, and AZC further inhibited this process. To further understand the effects of DNA methylation on TFEB expression, we used AZC to intervene in Hcy-induced hepatocytes and found increased TFEB mRNA and protein levels ( Figure 3D), suggesting that Hcy downregulates TFEB DNA methylation and upregulates TFEB expression in hepatocytes.

### DNMT3b positively modulates Hcy-induced TFEB DNA methylation in hepatocytes

**Figure 4:**
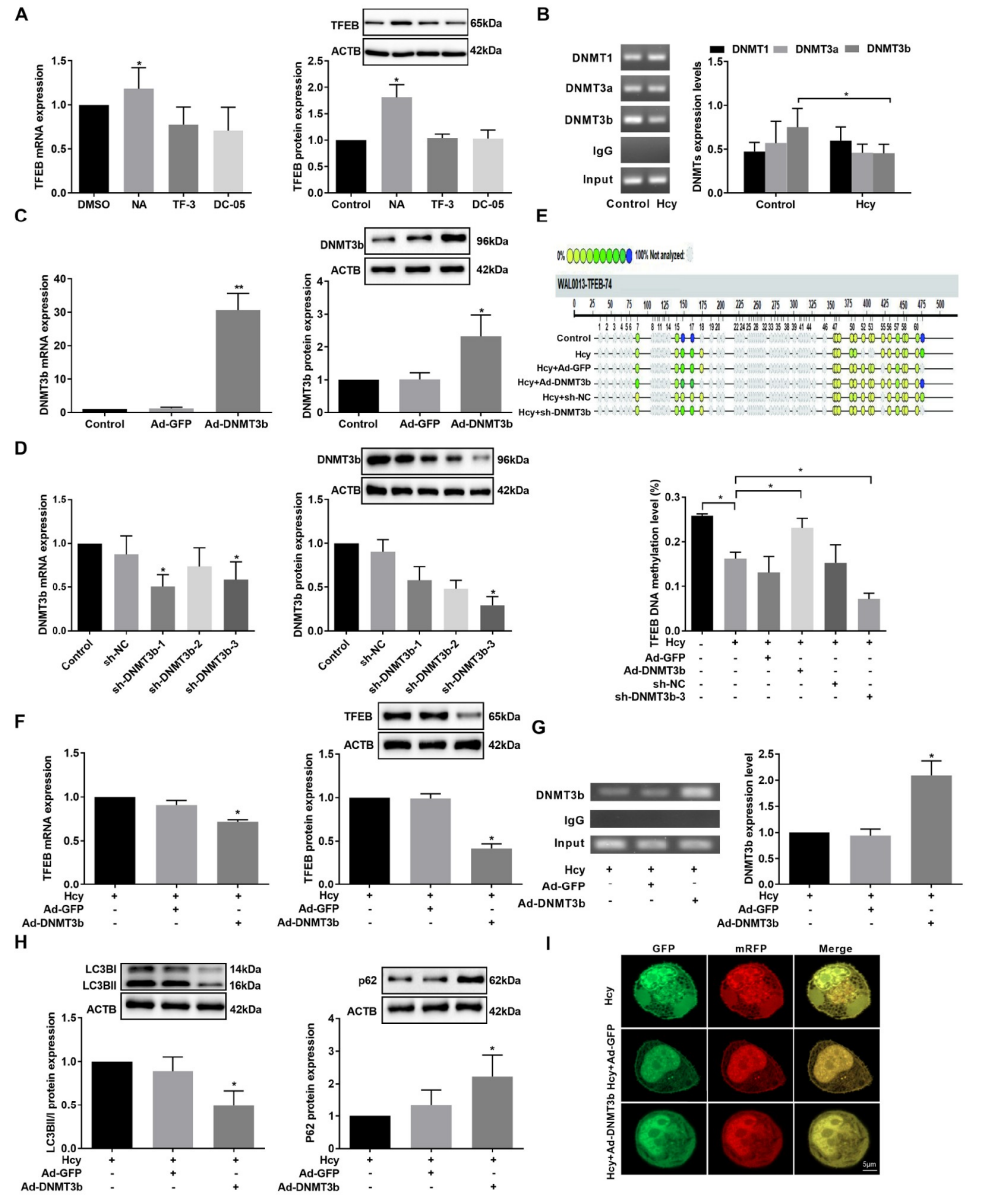
Figure 4 The effect of DNMT3b on TFEB DNA methylation and autophagy (A) The difference in TFEB mRNA and protein levels in hepatocytes exposed to Hcy and DC-05, TF-3, and nanomycin A (NA), the corresponding inhibitors of DNMT1, DNMT3a and DNMT3b, respectively. (B) The binding level between DNMT and the TFEB promoter in hepatocytes was examined by ChIP assay. (C) DNMT3b mRNA and protein levels were measured by qRT-PCR and western blot analysis, respectively in hepatocytes with DNMT3b adenovirus infection. (D) DNMT3b levels were measured by qRT‒PCR and western blot analysis in hepatocytes infected with sh-DNMT3b adenovirus (sh-DNMT3b-1, sh-DNMT3b-2, and sh-DNMT3b-3). (E) Quantitative analysis of DNA methylation of the TFEB promoter in hepatocytes after DNMT3b overexpression or inhibition. (F) The effect of DNMT3b overexpression on TFEB level in hepatocytes. (G) The binding level of DNMT3b to the TFEB promoter was measured by ChIP assay in hepatocytes exposed to Ad-DNMT3b. (H) Detection of LC3BII/I and p62 protein levels after overexpression of DNMT3b. (I) Confocal images of mRFP-GFP-LC3 fluorescent puncta in hepatocytes after exposure to Ad-DNMT3b (scale bar: 5 μm). Data are presented as the mean±SD from three independent experiments, * P<0.05, ** P<0.01.

To understand the role of DNMTs in regulating TFEB DNA methylation in hepatocytes, cells were exposed to DC-05, TF-3, and NA, which are DNMT1, DNMT3a and DNMT3b inhibitors, respectively. Interestingly, NA significantly increased TFEB expression in hepatocytes compared to other inhibitors ( Figure 4A). ChIP assay further demonstrated that DNMT3b, but not DNMT1 or DNMT3a, bound less to the proximal promoter region of *TFEB* after exposure to Hcy than in the control group in hepatocytes ( Figure 4B).

To gain insight into the role of DNMT3b in the regulation of DNA methylation in the *TFEB* promoter, we infected hepatocytes with DNMT3b-overexpressing adenovirus (Ad-DNMT3b) and detected the expression of DNMT3b. The data showed that DNMT3b mRNA and protein levels were increased ( Figure 4C). Meanwhile, we screened the most functional DNMT3b interference adenovirus (sh-DNMT3b) and verified its efficiency. Remarkably, sh-DNMT3b-1 showed the best interference efficiency ( Figure 4D). Overexpression of DNMT3b upregulated TFEB DNA methylation and inhibited its RNA and protein expression, while knockdown of *DNMT3b* further inhibited TFEB DNA methylation in Hcy-induced hepatocytes ( Figure 4E,F). In addition, overexpression of DNMT3b increased its binding efficacy in the *TFEB* promoter and reduced autophagy in hepatocytes ( Figure 4G,H). Finally, using mRFP-GFP-LC3 adenovirus, we found that autophagosomes and autophagic lysosomes were decreased in DNMT3b-overexpressing hepatocytes exposed to Hcy ( Figure 4I). Taken together, DNMT3b is a specific methyltransferase that regulates TFEB methylation and autophagy in Hcy-induced hepatocytes.

## Discussion

In the present study, we examined the role of TFEB in Hcy-induced hepatocyte autophagy. Our findings confirmed the role of DNA hypomethylation in upregulating TFEB expression, which leads to Hcy-induced autophagy in hepatocytes.

Hcy is a sulfur-containing amino acid produced in internal metabolism [19]. As the liver is one of the principal organs of Hcy metabolism, once liver function is impaired, abnormal methionine metabolism occurs, which leads to the release of accumulated Hcy into the plasma. Hcy, in turn, can affect liver function. Hcy has been linked to the pathogenesis of several diseases, including coronary artery disease and liver disease, both of which are characterized by elevated levels of total Hcy in plasma [20]. In the early stage, the HHcy model of ApoE ^‒/‒^ mice was replicated by feeding with a high methionine diet, and serum Hcy was detected to verify model establishment [21]. We also found that HHcy caused liver damage in ApoE ^‒/‒^ mice, and Hcy increased the level of autophagy in the liver tissue of ApoE ^‒/‒^ mice.

TFEB is a member of the leucine zipper family of transcription factors. It was found that TFEB nuclear translocation increased the transcription of genes encoding autophagic and lysosomal proteins, thereby promoting lysosomal biogenesis and autophagosome formation and increasing autophagy [22]. Moreover, ethanol activation of mTORC1 disrupts TFEB-mediated liver lysosomal biogenesis, resulting in autophagy deficiency in mice. In contrast, overexpression of TFEB increases the biogenesis of lysosomes and prevents ethanol-induced steatosis and liver injury [23]. These studies suggest that liver autophagy and lysosome function can be regulated by changing the activity of TFEB. We found that increased TFEB boosted autophagy in Hcy-induced hepatocytes. Conversely, knockdown of *TFEB* decreased this effect.

Elevated Hcy regulates genomic DNA methylation and induces specific methylation in the promoter regions of genes associated with human diseases, including carcinoma, mental illness, neurodegenerative disease, and cardiovascular disease [ 24‒ 26] . DNA methylation is a biological process in which a methyl group is covalently linked to cytosine to produce 5-methylcytosine (5mC) [27]. DNA methylation has been widely considered an epigenetic silencing approach that plays a role in numerous cellular metabolic events, including X-chromosome inactivation, genomic imprinting, and gene transcription [28]. The methylation process is catalyzed by a group of enzymes called DNMTs. DNMT3a and DNMT3b are responsible for the *de novo* methylation mode, whereas DNMT1 is responsible for maintaining methylation. Therefore, Hcy is an important intermediate that plays a crucial role in DNA methylation [29].

Numerous studies have demonstrated that methylated sequences are not recognized by transcription factors, which prevents the expression of corresponding genes [30]. DNA methylation of cytosine residues transforming to 5-methylcytosine may assist in this process, so the regulatory regions of these genes are frequently hypomethylated, which results in gene overexpression, or hypermethylated, which silences these genes [31]. The methyl group used for methylation reactions originates from S-adenosylmethionine (SAM), an intermediate in the metabolism of Hcy. Upon methylation, SAM is converted to S-adenosylhomocysteine (SAH), which inhibits transmethylation reactions. HHcy could lead to global hypomethylation through SAH aggregation and decreased methylation capacity indicated by decreased SAM/SAH ratio [32]. Ma *et al*. [33] found that Hcy induces extracellular-superoxide dismutase (EC-SOD) DNA hypomethylation in macrophages and that DNMT1 acts as the essential enzyme in the methyl transfer process, leading to downregulation of EC-SOD expression and increased atherosclerosis. In this study, we found that the level of TFEB DNA methylation is decreased in Hcy-incubated hepatocytes, and Hcy mainly affects DNMT3b to upregulate TFEB expression. Using an adenovirus overexpressing DNMT3b, we found that the methylation levels of TFEB were elevated, the expression of TFEB was decreased, and the level of hepatocyte autophagy was suppressed.

**Figure 5:**
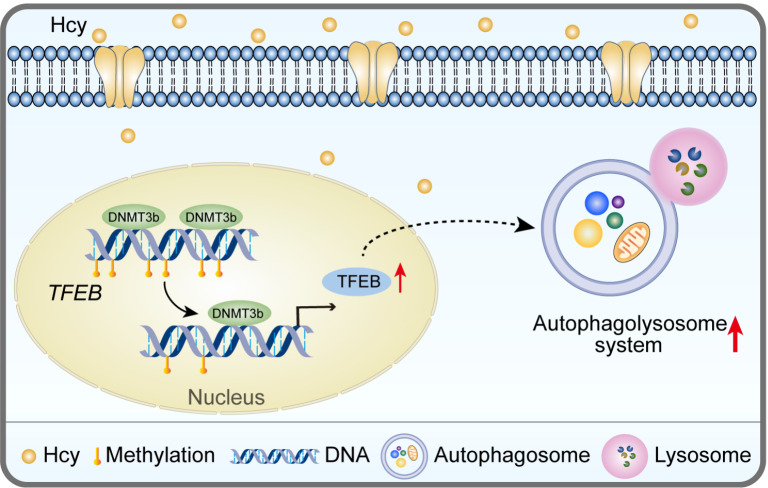
Figure 5 Homocysteine accelerates hepatocyte autophagy by upregulating TFEB via DNMT3b-mediated DNA hypomethylation TFEB is an important regulatory mediator of autophagy, and its expression is modulated by DNA methylation.

In conclusion, our data revealed that TFEB plays a key role in Hcy-induced hepatocyte autophagy. Meanwhile, TFEB DNA hypomethylation upregulates TFEB expression, which is a new mechanism by which Hcy promotes hepatocyte autophagy ( Figure 5). Nevertheless, the potential role of TFEB in Hcy-induced autophagy needs to be further investigated.
